# Anterior Scleral Regional Variation between Asian and Caucasian Populations

**DOI:** 10.3390/jcm9113419

**Published:** 2020-10-25

**Authors:** Alejandra Consejo, Richard Wu, Ahmed Abass

**Affiliations:** 1 Institute of Physical Chemistry, Polish Academy of Sciences, 01-224 Warsaw, Poland; alejandra.consejo@ichf.edu.pl; 2 Department of Applied Physics, University of Zaragoza, 50009 Zaragoza, Spain; 3 Department of Optometry, Central Taiwan University of Science and Technology, Taichung City 40601, Taiwan; richard.wu@brightenoptix.com; 4 College of Optometry, Pacific University, Forest Grove, OR 97116, USA; 5 School of Engineering, University of Liverpool, Liverpool L69 3GH, UK

**Keywords:** anterior eye surface, sclera, ethnicity, topography, profilometry

## Abstract

Purpose: To evaluate the anterior scleral shape regional differences between Asian and Caucasian populations. Methods: The study included 250 Asian eyes and 235 Caucasian eyes from participants aged 22 to 67 years (38.5 ± 7.6). Three-dimensional (3D) corneo-scleral maps were acquired using a corneo-scleral topographer (Eye Surface Profiler, Eaglet Eye BV) and used to calculate sagittal height. For each 3D map, the sclera (maximum diameter of 18 mm) and cornea were separated at the limbus using an automated technique. Advanced data processing steps were applied to ensure levelled artefact-free datasets to build an average scleral shape map for each population. Results: Statistically, Asian and Caucasian sclerae are significantly different from each other in sagittal height (overall sclera, *p* = 0.001). The largest difference in sagittal height between groups was found in the inferior-temporal region (271 ± 203 µm, *p* = 0.03), whereas the smallest difference was found in the superior-temporal region (84 ± 105 µm, *p* = 0.17). The difference in sagittal height between Caucasian and Asian sclera increases with the distance from the limbus. Conclusions: Asian anterior sclera was found to be less elevated than Caucasian anterior sclera. However, the nasal area of the sclera is less elevated than the temporal area, independently of race. Gaining knowledge in race-related scleral topography differences could assist contact lens manufacturers in the process of lens design and practitioners during the process of contact lens fitting.

## 1. Introduction

Contact lenses are a popular form of vision correction as prescriptions of contact lenses increase worldwide on a yearly basis [1,2,3]. One of the main reasons for this constant rise is the progressive increase in the number of people in need of vision correction due to myopia, which widely affects Asian populations [3].

Even though disposable soft contact lenses are still the preferred option among users [2,4,5], scleral contact lenses are gaining great interest as an alternative solution for vision correction [6,7], especially in compromised eyes [6]. As scleral lenses became more popular, practitioners and researchers gained interest in describing scleral morphometry accurately [8,9,10,11] and investigating how the corneo-scleral area is affected as a consequence of contact lens wear [12,13,14]. As the Asian population and Caucasian population markets are forming the biggest markets in the world [3], the differences between the eyes of these populations need to be identified.

Even though some contact lens manufacturers have specific lens designs for Asian eyes [15], designers of contact lenses are often discouraged to know why some of their lenses are successfully working with a particular set of fitting rules with a specific population, but they have to change their fitting rules or even their design when they try to fit their lenses to customers in a new market dominated by a different ethnic group [16,17]. In this context, in a previous work by Vincent and colleagues, differences in the ocular response to scleral lens wear were observed between Asian and Caucasian eyes [18].

To overcome this limitation and to better understand contact lens discomfort, race-related differences in ocular surface integrity have been investigated [19,20,21,22,23,24,25]. Specifically, differences in tear film stability [20] and tear film break up between Asian and Caucasian eyes have been reported [21]. Similarly, race-related differences in visual axis [22] and ocular anatomy, including eyelids [23] and corneal shape [23,24,25], have been acknowledged. However, despite its importance for a successful scleral lens fit, precise race-related differences in scleral shape are not yet available. Even though contact lens fitting is an individualized procedure, particularly in scleral lens wear where the scleral topography can vary substantially between the two eyes of an individual, gaining knowledge on race-related differences in scleral shape could be of use to those practitioners who do not have access to a corneoscleral topographer in their practice.

The current study presents a comprehensive comparison between the anterior scleral shape of Asian and Caucasian populations. Advanced data processing steps were applied to ensure levelled artefact-free datasets to build an average scleral shape map for each population.

## 2. Materials and Methods

### 2.1. Participants

In this record review study, both right and left eye anonymized topography data were extracted from the recorded data of 125 Taiwanese Asian (250 eyes) and 118 Caucasian (235 eyes) participants age-matched from 22 to 67 years (38.5 ± 7.6), independent *t*-test *p* = 0.56. Groups were properly gender-balanced (Asians: 66 females (52.8%) and 59 males; Caucasians: 63 females (53.4%) and 55 males). No participant had been recruited specially for this study, so fully anonymized secondary data were used. The study utilized a collection of clinical data that has been used in various previous studies [8,26,27,28,29], where only healthy eyes were selected to be processed. Potential participants with corneal abnormalities were not included in the study. Exclusion criteria also included the presence of any conjunctival or scleral pathology. Data presented in the current work were collected from two different clinical sites (The University of Manchester (UK), and the Brighten Optix Corporation (Taiwan)). Practitioners responsible for data acquisition in both clinical settings were experienced clinicians, accustomed to working with ESP. Recorded data for individuals who were suffering from ocular diseases or having a history of trauma or ocular surgery, including Asian upper blepharoplasty, were excluded. According to the University of Liverpool’s Policy on Research Ethics, ethical approval was unnecessary for secondary analysis of fully anonymized data. The study followed the tenets of the Helsinki Declaration.

Participants were told not to wear contact lenses for 1 week before the topography measurement [13]. The eye surface scan process was carried out using a non-contact corneo-scleral topographer (Eye Surface Profiler (ESP), Eaglet Eye BV, AP Houten, The Netherlands), a height profilometer with the potential to measure the corneo-scleral topography far beyond the limbus [30]. Accurate measurements of anterior eye surface using ESP require the instillation of fluorescein with a more viscous solution than saline [30]. The Bio-Glo (HUB Pharmaceuticals; www.hubrx.com/) ophthalmic strips were used to touch the eye’s upper and lower fornixes gently. They were impregnated with 1 mg of fluorescein sodium ophthalmic moisten with one drop of an eye lubricant (HYLO-Parin or Lubristil, 1 mg/mL of sodium hyaluronate). Participants were asked to put their chin on the headrest of the ESP device and focus on the internal instrument’s target. The operator had to align the instrument until sufficiently good image quality, indicated by the device, was achieved. Participants were instructed to open their eyes wide prior to the ESP measurements to ensure surface data coverage up to a few millimeters beyond the limbal zone. Measurements in which eyelids covered the corneo-scleral area were excluded. From the three measurements acquired per eye, the one with the largest scleral area coverage was included for data analysis. It is important to highlight that to avoid bias, right and left eyes were always treated independently from each other, and no merging data technique was applied in this work. To investigate the difference between right and left eyes among the same population, left eyes were flipped to avoid mirror asymmetry (e.g., nasal part of a right eye would coincide with the nasal part of a left eye).

### 2.2. Data Exportation and Processing

The data was exported from the ESP software in MATLAB^®^ (MathWorks, Natick, MA, USA) binary data container format (*.mat) where the characteristics of eyes, as measured by the ESP system, were extracted and processed. The eye surface data was processed by custom-built MATLAB codes entirely independent from the built-in ESP software digital signal processing (DSP) algorithms.

In order to make valid comparisons, three main data processing steps were followed for each measurement: (1) data orientation, (2) removal of outliers, and (3) interpolation.

Even though the instrument has an internal procedure for visual axis alignment, extra calculations to ensure that all eyes share the same orientation might be necessary [27,28]. It is also known that fixation on a short distance object like the ESP target needs a response from the human ocular system to accomplish a focused image [31]. As the foveal center, the sensitive part of the retina, is located approximately 3.4 mm temporal to the optic disk boundary [32], 2.5 mm temporal to the eye’s optical axis [33] and slightly inferior, the eye tends to rotate to a tilted position to direct the refracted light rays to drop on the fovea. To overcome this circumstance, eyes were treated individually. First, the limbus profile of each eye was located using the 3D non-parametric method presented in a previous study [27]. Further, each eye’s topography data was levelled to the best-fit plane that passed through the detected limbus. To achieve this levelling, the angles of the limbus plane with the horizontal and vertical axis $\alpha_{x}$ and $\alpha_{y}$ were determined by the inverse trigonometric cosine function of the dot product of the normal vector of the limbus plane $\left(N_{x},N_{y},N_{z}\right)$ and each of the *Y*-axis (0,1,0) and *X*-axis (0,0,1) unit vectors respectively as presented in Equations (1) and (2).

(1) $$\alpha_{x}=\frac{-\pi }{2}+\cos^{-1}\left(\left(N_{x},N_{y},N_{z}\right)\cdot \left(0,1,0\right)\right)$$

(2) $$\alpha_{y}=\frac{-\pi }{2}+\cos^{-1}\left(\left(N_{x},N_{y},N_{z}\right)\cdot \left(0,0,1\right)\right)$$

Then eye surface was rotated around the X-axes and Y-axes by the tilt angles $\alpha_{x}$ and $\alpha_{y}$, respectively, in order to level each eye’s limbus plane in the XY-plane. The 3D rotation was achieved by applying 3D rotation matrices [34].

(3) $$R_{x}\left(\alpha_{x}\right)=\left[\begin{array}{ccc} 1 & 0 & 0\\ 0 & \cos\alpha_{x} & -\sin\alpha_{x}\\ 0 & \sin\alpha_{x} & \cos\alpha_{x} \end{array}\right]$$

(4) $$R_{y}\left(\alpha_{y}\right)=\left[\begin{array}{ccc} \cos\alpha_{y} & 0 & \sin\alpha_{y}\\ 0 & 1 & 0\\ -\sin\alpha_{y} & 0 & \cos\alpha_{y} \end{array}\right]$$

(5) $$R_{z}\left(\alpha_{z}\right)=\left[\begin{array}{ccc} \cos\alpha_{z} & -\sin\alpha_{z} & 0\\ \sin\alpha_{z} & \cos\alpha_{z} & 0\\ 0 & 0 & 1 \end{array}\right]=\left[\begin{array}{ccc} 1 & 0 & 0\\ 0 & 1 & 0\\ 0 & 0 & 1 \end{array}\right]$$

where $\alpha_{x}$, $\alpha_{y}$, and $\alpha_{z}$ were the rotating angles in *X*, *Y,* and *Z* directions, respectively. As Equation (5) indicates, the rotation angle about the *Z*-axis, $\alpha_{z}$, was set to zero [12].

Following the elemental rotation rule, the rotated coordinates of the corneal surface $x_{rn}$, $y_{rn}$ and $z_{rn}$ were calculated as

(6) $$\left[\begin{array}{ccccc} x_{r1} & x_{r2} & x_{r3} & \ldots & x_{rn}\\ y_{r1} & y_{r2} & y_{r3} & \ldots & y_{rn}\\ z_{r1} & z_{r2} & z_{r3} & \ldots & z_{rn} \end{array}\right]=\left[R_{x}\left(\alpha_{x}\right)R_{y}\left(\alpha_{y}\right)R_{z}\left(\alpha_{z}\right)\right]\left[\begin{array}{ccccc} x_{1} & x_{2} & x_{3} & \ldots & x_{n}\\ y_{1} & y_{2} & y_{3} & \ldots & y_{n}\\ z_{1} & z_{2} & z_{3} & \ldots & z_{n} \end{array}\right]$$

where $x_{n}$, $y_{n}$, and $z_{n}$ are the original coordinates before rotation and *R_x_*, *R_y_*, and *R_z_* are the rotational matrices (Equations (3)–(5)).

Before moving to the next processing stage, the origin position (0,0,0) of each levelled eye’s surface was shifted to the highest point (corneal apex) after levelling.

After applying this procedure, which would ensure that all eyes are equally oriented, the following step was outlier removal where artificial edges around each eye’s profile were removed. The artefacts removing strategy was based on the observation that the artefacts in the measured eye surface do not follow the natural shape of the eye [29]. The sudden lift or sharp descent usually existing in the measured eye surface were effects of tears, eyelid edges or lashes appearing. Using the principles of robust statistics, which are not unduly affected by outliers, edge-effects were detected by calculating the moving median of the eye height data along eye meridians [29].

Finally, after data extraction and preparation, sagittal height was calculated. To this end, all exported eyes were interpolated, using 3D triangulation-based fitting, to a mesh-grid of 201 points in the X direction and 201 in the Y direction, giving in total 40,401 data points per eye covering a range from −10 to 10 mm in steps of 0.1 mm in both X and Y directions. After this process, all the interpolated eyes’ data shared the exact X and Y coordinates, however, each eye had its own height or Z-coordinate (equivalent to sagittal height). At that point, the eyes’ height data for each population were averaged, and the standard deviation was calculated per each data point. No extrapolation techniques were used; therefore, anterior eye surface points with no values were excluded from determining the mean and the standard deviation values. Those eyes that did not reach at least 85% of scleral coverage for a given diameter were not considered for statistical analysis. It is worth noting that in the current work, intuitive terms as ‘flatter’ and ‘steeper’ are used to describe scleral shape even though curvature maps were not available from ESP. Sagittal height (elevation) maps were analyzed instead. However, from a sagittal height, it is possible to infer curvature-related information. For a given chord, a smaller elevation corresponds to a flatter surface, and a larger elevation corresponds to a steeper surface.

In addition to corneoscleral maps, the value of the corneal sphere, expressed in dioptres, and available from ESP software, was exported and utilized to estimate the refractive state of participants. The statistical analysis was performed using SPSS statistics software version 25.0 (SPSS Inc., Chicago, IL, USA). The null hypothesis, at 95.0% confidence level testing, was used to investigate the inferences of the findings based on statistical evidence. Normality of all data sets was not rejected (Shapiro–Wilk test, *p* > 0.05). Furthermore, the ANOVA-repeated-measurements test (adjustment for multiple comparisons: Bonferroni) was performed to ascertain whether there was a difference in sagittal height depending on the diameter considered and the angular position. The race was considered a between-subjects factor. Corresponding results are presented as F (degrees of freedom, the error of degrees of freedom) along with the corresponding *p*-value and partial eta squared (ƞ^2^), which is a measure of effect size. Post-hoc comparisons are also reported. Mauchly’s test of sphericity indicated that the assumption of sphericity had not been violated in any of the parameters under analysis. Further, the two-sample paired *t*-test was applied to investigate whether there was a statistically significant difference between right and left eyes. In addition, an independent *t*-test was applied to investigate whether the groups were matched in terms of refractive state (corneal sphere). As topography readings between right and left eyes of a healthy subject are highly correlated, eyes of the same subject were treated separately and not combined for statistical analysis. In the Results section, findings for left eyes only are reported except where otherwise stated.

## 3. Results

**Figure 1:**
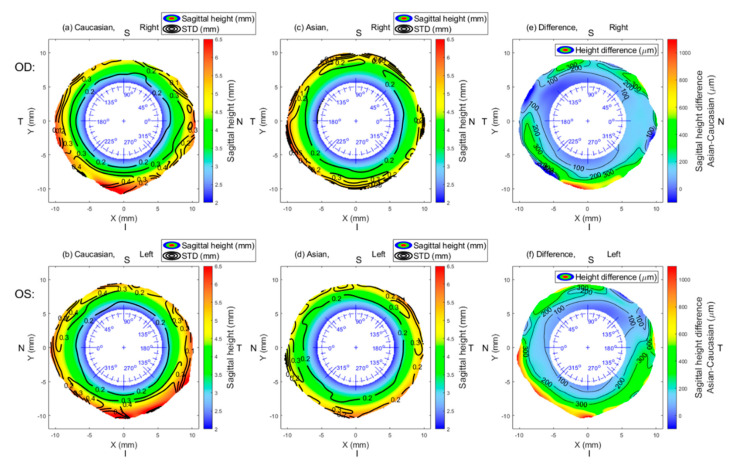
Mean sagittal height of Asian eyes (OD, *n* = 125; OS, *n* = 125) (**a**,**b**) and Caucasian eyes (OD; *n* = 114, OS; *n* = 121) (**c**,**d**) and the mean group difference (**e**,**f**). Contour lines in subfigures (**a**–**d**) represent the standard deviation. In subfigures (**e**) and (**f**), contour lines join the same numerical values of the difference. N: nasal, I: inferior, T: temporal, S: superior.

**Figure 2:**
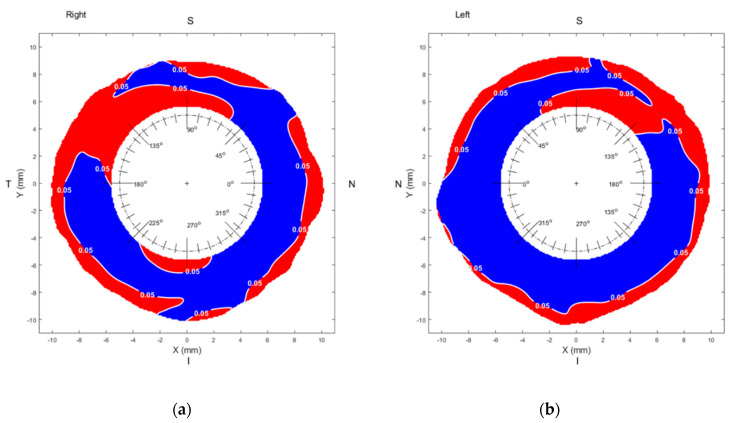
Statistical significance map between Asian (OD, *n* = 125; OS, *n* = 125) and Caucasian populations (OD, *n* = 114; OS, *n* = 121). The border between significance (blue) and non-significance (red) is plotted as a white contour line at *p* = 0.05. N: nasal, I: inferior, T: temporal, S: superior. (**a**) Right eye, (**b**) Left eye.

Asian and Caucasian sclerae are different from each other in sagittal height (overall sclera, *p* = 0.001). Asian sclera was found to be flatter than Caucasian sclera. Table 1 and Table 2 show the mean group values of sagittal height for the two groups under investigation, while Table 3 shows the differences in sagittal height between both groups. Global group mean maps are shown in Figure 1. Even though the sagittal height of Caucasians was found to be higher than that of Asians, this difference was not always significantly different, as indicated by Figure 2.

Considering the overall sclera (from 6–9 mm annulus), the largest difference between Asian and Caucasian sclera was found in the inferior-temporal region (271 ± 203 µm, *p* = 0.03), whereas the smallest difference was found in the superior-temporal region (84 ± 105 µm, *p* = 0.17).

Sagittal height was found to depend on the angular orientation, independently of race, F (7, 98.8) = 23.7, *p* < 0.001, ƞ^2^ = 0.14. However, considering the race as a between-subjects factor, it was also observed that the difference between Caucasian and Asian sclera depends on the angular position (Table 3), F (7, 98.8) = 3.14, *p* = 0.003, ƞ^2^ = 0.02. All pairwise comparisons between angular positions were statistically significant, except for N vs. I-N (both eyes, *p* > 0.05) and S vs. S-N (both eyes, *p* > 0.05). Thus, regarding the main meridians (superior, inferior, nasal, and temporal), statistically significant local differences were found between Asian and Caucasian sclerae.

Independently of race, considering the overall sclera (from 6–9 mm annulus), the nasal region (Asians: 3.33 ± 0.72 mm; Caucasians: 3.50 ± 0.79 mm) was found to be less elevated than the temporal region (Asians: 3.55 ± 0.89 mm; Caucasians: 3.77 ± 0.95 mm). The difference in sagittal height between the nasal and temporal region was statistically significant for both groups (Asians: *p* = 0.04; Caucasians: *p* = 0.04). On the other hand, the superior regions (Asians (from 6–9 mm annulus): 3.59 ± 0.84 mm and Caucasians (from 6–8 mm annulus): 3.30 ± 0.68 mm) and inferior regions (Asians (from 6–9 mm annulus): 3.60 ± 0.87 mm and Caucasians (from 6–8 mm annulus): 3.81 ± 0.99 mm) were not found to be statistically significant from each other independently of race (Asians: *p* = 0.72; Caucasians: *p* = 0.14).

Sagittal height was found to increase with the distance from the limbus, independently of race, F (3, 8.1) = 5115, *p* < 0.001, ƞ^2^ = 0.98. However, considering the race as a between-subjects factor, it was also observed that the difference between Caucasian and Asian sclera increments with the distance from the limbus (Table 3), F (3, 8.1) = 10.0, *p* < 0.001, ƞ^2^ = 0.08. All pairwise comparisons between radii were statistically significant (both eyes, all *p* < 0.001). It was observed that the same way the group mean value increments with distance from limbus, so does the standard deviation (Table 3), suggesting substantial inter-subject variation in sagittal height with the distance from limbus, independently of race.

In general, the right and left eyes were found to be not significantly different from each other. This applies to all the chords and regions under analysis for Caucasian eyes (Table 4 left). In Asian eyes, however, a statistically significant difference between right and left eyes was found in the nasal area (Table 4 right).

The corneal sphere of Caucasians (43.7 ± 1.7) was not found to be statistically significantly different from that of Asians (43.5 ± 1.6) (two-sample independent *t*-test, *p* = 0.25). Similarly, no statistically significant differences were found between right and left eyes in the corneal sphere of Caucasians (paired *t*-test, *p* = 0.86) nor Asians (paired *t*-test, *p* = 0.45).

In regards to scleral coverage, as Table 5 indicates, the larger the diameter, the smaller number of eyes reached full coverage of the scleral area. This affected both races, especially in the superior region. Those eyes that covered at least 85% of scleral average in a given diameter were considered as acceptable and included for statistical analysis (Figure 1 and Figure 2, Table 1 and Table 4).

## 4. Discussion

To the best of our knowledge, this is the first study to define race-related differences in the shape of the human anterior sclera. From a total of 435 ocular 3D corneo-scleral topographic maps of 125 Asians and 118 Caucasians, the study described the mean elevation of the human sclera and found that the Asian sclera is overall flatter than Caucasian sclera.

For both ethnicities, the average elevation for a from 6–9 mm scleral annulus was higher for the temporal sclera and lower for the nasal sclera, in accordance with previous works based on Asian [35,36] and Caucasian participants [8,9,37]. Similarly, and also following previous literature [8,35], no significant difference was found between superior and inferior sectors for any of the groups.

Even though Asian and Caucasian sclerae seem to follow a common pattern, significant statistical differences between the sclera of Asian and Caucasian eyes were frequently found (Table 3 and Figure 2). In particular, the largest difference between groups was found in the inferior-temporal sector. Race-related differences in the eyelids [38] or refractive power [3] could justify the observed differences between Asian and Caucasian sclera. Asian eyelids differ from Caucasian eyelids in several features, such as low, poorly defined lid creases; pronounced fullness of the upper and lower lids; narrower palpebral fissures; and common presence of epicanthal folds [39]. Eyelids are in close contact with the ocular surface exerting pressure on it [40]. Consequently, differences in eyelid anatomy could potentially lead to differences in ocular topography. Likewise, as indicated by Table 4, a statistically significant difference was found in the nasal area between right and left Asian sclerae. Palpebral fissure asymmetry is more common among Asian [41] than Caucasian eyes [42]. This complicates ptosis surgery [43] since postoperative asymmetry, the most common source of dissatisfaction following Asian upper blepharoplasty, is more common when eyelid asymmetry exists preoperatively [43]. Gaining knowledge of the shape of the Asian sclera could help the surgeon avoid undesired surgical outcomes. Regarding differences in refractive power, Asian populations are more prone to myopia than Caucasians [3]. A previous study on corneo-scleral topography showed that scleral shape is highly correlated with axial length (r = 0.76, *p* < 0.001) and moderately correlated with refractive power (r = 0.48, *p* < 0.01) [44]. The more myopic an eye is the flatter the anterior sclera [44,45]. This finding agrees with the fact that in the current work, the sclera of Asians was found to be flatter than that of Caucasians. Similarly, in that same previous work, the largest difference between myopes and emmetropes was found in the temporal and inferior temporal sector [44], which coincides with the differences reported between Asian and Caucasian sclerae. A limitation of the current study is that the refractive power of the participants was not measured. To overcome this limitation, the value of the corneal sphere (equivalent to the corneal radius of curvature), available from ESP software, was investigated. No statistically significant difference was found in the corneal sphere (D) of Asians and Caucasians. According to a previous work based on a cohort of over 6000 eyes, corneal radius and spherical equivalent of the eye are strongly correlated with each other (r = 0.71, *p* < 0.001) [46]. This previous finding suggests that, since both groups in the current work were matched in terms of corneal power, the mean refractive error in each group would likely be matched. However, whether the generally observed refractive error differences between Caucasian and Asian populations lay on axial length, as it was traditionally considered [47], or rather on corneal radius, ref. [48] seems to be undefined [23]. Due to this controversy, the current work does not provide sufficient data to make a strong statement regarding the origin of the observed differences. Consequently, the reported findings should be confirmed in an emmetropic cohort of participants, where both axial length and refractive error would be measured, in addition to corneoscleral topography.

During ESP data acquisition, fluorescein is required to cover the eye surface. This process might be affected by the quality of the tear film, as indicated by Garaszczuk and Iskander [49]. Even though the stability of the tear film could be different between ethnicities [15], there was no record of significant alterations in the tear film of any of the participants. Even though the data used in the current study was retrospective, the participants underwent a complete ophthalmological examination, including tear film stability, before being classified as ‘healthy’ in the database of the different participating clinical sites. Consequently, we would not expect tear film stability to affect the result presented in the current work. In a similar manner, even though the conjunctiva is known to evolve with time, changing its thickness as the eye ages [50], we would not expect this to alter the results presented in the current work. Firstly, because in both racial groups, the participants were age-matched, and secondly because the average thickness of the conjunctiva is of the same order of magnitude than the resolution of the measuring device, especially in the peripheral anterior sclera [30].

The current findings regarding the differences in scleral shape between Asian and Caucasian sclerae might be of use for contact lens manufacturers (contact lens peripheral zoon design) and also practitioners who do not count with the support of a corneoscleral topographer in their practice. The asymmetrical nature of the sclera and the limbus has been acknowledged as a contact-lens-fitting challenge [13], especially in speciality lens wear [9,51,52]. Likewise, larger amounts of scleral asymmetry were found to be correlated with more pronounced lens decentration [53]. Although the importance of scleral topography for an optimal contact lens fit has been recognized, traditionally, works in scleral topography have been restricted to assessing a few isolated scleral points [9,37]. However, in the current work, an artefact-free methodology based on continuous 3D data was applied, resulting in an accurate description of the Caucasian and Asian scleral shape. It is also worth mentioning that the sample size here used was significantly larger than that from previous works regarding scleral topography [8,9,35,36,37].

This study has some limitations concerning the coverage of the scleral area far beyond the limbus. As Table 5 indicates, a full coverage up to 18 mm was reached in a few eyes. The superior area was the most affected by limited scleral coverage, in agreement with the previous report from DeNaeyer et al. where they reported a decreasing scleral coverage with an increasing chord in a straight gaze image, using the sMap3D topographer [54]. In the current study, following the criteria from previous works on scleral characterisation, ref. [7] topographies with 15% or larger amount of missing data were not included in the analysis. Due to this scleral coverage limitation, peripheral results should be interpreted with caution. As with most clinical tests, participant cooperation was necessary during the eye test. Slight participant body movement may reduce the quality of the eye scan and leave the task of soothing the results for the scanning machine software. Generally, the shorter acquisition time, the fewer motion-related artefacts. Adding the fact that the quality of clinical measurements of an eye is also dependent on the performance of the operator of the machine, the influence of this factor reduces with single-shot shorter acquisition time eye scanners, such as those from ESP (few milliseconds [25]). From this angle, this study uses a fast single-shot corneo-scleral measurement for each eye to evaluate the difference between Asian and Caucasian populations, performed by experienced operators, which would potentially minimize bias in the measurements [25]. The study considered levelling eyes’ surfaces to the limbus and applied an advanced technique to calculate variances in sets of data that are free of edge-effect artefacts.

## 5. Conclusions

Full 3D scleral maps were used to accurately describe race-related differences in the shape of the human anterior sclera. The nasal area of the sclera is less elevated than the temporal area, independently of race. However, overall, the Asian anterior scleral was found to be flatter than Caucasian anterior sclera.

## Figures and Tables

**Table 1:** Mean sagittal height of the sclera for Caucasian eyes (*n* = 121) for a diameter of 12 mm, 14 mm, 16 mm, and 18 mm. Sagittal height is expressed in mm. In brackets, the standard deviation.

Angular position			**Distance from the Corneal Centre**			
			**6 mm**	**7 mm**	**8 mm**	**9 mm**
	0°	N	2.43 (0.14)	3.16 (0.20)	3.84 (0.23)	4.55 (0.28)
	45°	S-N	2.46 (0.17)	3.18 (0.24)	3.95 (0.31)	4.78 (0.40)
	90°	S	2.47 (0.17)	3.31 (0.21)	4.18 (0.26)	5.07 (0.29)
	135°	S-T	2.46 (0.16)	3.28 (0.22)	4.16 (0.27)	5.07 (0.32)
	180°	T	2.49 (0.16)	3.36 (0.22)	4.21 (0.25)	5.03 (0.20)
	225°	I-T	2.55 (0.17)	3.40 (0.20)	4.28 (0.29)	5.29 (0.48)
	270°	I	2.52 (0.14)	3.33 (0.20)	4.19 (0.33)	5.20 (0.46)
	315°	I-N	2.50 (0.14)	3.22 (0.18)	3.95 (0.26)	4.89 (0.38)

**Table 2:** Mean sagittal height of the sclera for Asian eyes (*n* = 125) for a diameter of 12 mm, 14 mm, 16 mm, and 18 mm. Sagittal height is expressed in mm. In brackets, the standard deviation.

Angular position			**Distance from the Corneal Centre**			
			**6 mm**	**7 mm**	**8 mm**	**9 mm**
	0°	N	2.34 (0.13)	3.04 (0.18)	3.68 (0.21)	4.27 (0.28)
	45°	S-N	2.39 (0.13)	3.08 (0.17)	3.75 (0.20)	4.42 (0.26)
	90°	S	2.46 (0.13)	3.20 (0.17)	3.99 (0.23)	4.71 (0.27)
	135°	S-T	2.44 (0.12)	3.24 (0.18)	4.02 (0.28)	4.93 (0.35)
	180°	T	2.35 (0.13)	3.16 (0.20)	3.97 (0.25)	4.73 (0.36)
	225°	I-T	2.41 (0.13)	3.20 (0.18)	3.97 (0.21)	4.85 (0.26)
	270°	I	2.46 (0.13)	3.19 (0.17)	3.93 (0.22)	4.82 (0.27)
	315°	I-N	2.41 (0.14)	3.07 (0.18)	3.71 (0.24)	4.40 (0.25)

**Table 3:** Mean sagittal height difference (Caucasian–Asian) for a population of 125 Asian eyes and 121 Caucasian eyes for a diameter of 12 mm, 14 mm, 16 mm, and 18 mm. The sagittal height difference is expressed in µm. In brackets, the standard deviation.

Angular position			**Distance from the Corneal Centre**			
			**6 mm**	**7 mm**	**8 mm**	**9 mm**
	0°	N	88 (57)	128 (91)	157 (96)	280 (24)
	45°	S-N	63 (116)	94 (171)	199 (238)	360 (298)
	90°	S	13 (111)	112 (110)	194 (124)	368 (120)
	135°	S-T	21 (111)	37 (138)	133 (33)	144 (137)
	180°	T	142 (94)	196 (94)	246 (45)	299 (297)
	225°	I-T	136 (110)	203 (99)	306 (200)	438 (404)
	270°	I	59 (46)	142 (102)	255 (250)	380 (372)
	315°	I-N	91 (0)	150 (27)	241(92)	493 (289)

**Table 4:** *p*-values (paired two-sample *t*-test) that compare the sagittal height of right and flipped left eye for Caucasian (left) and Asian (right) groups for a diameter of 12 mm, 14 mm, 16 mm, and 18 mm.

Angular position			**Caucasians**						**Asians**			
			**Distance from the Corneal Centre**						**Distance from the Corneal Centre**			
			**6 mm**	**7 mm**	**8 mm**	**9 mm**			**6 mm**	**7 mm**	**8 mm**	**9 mm**
	0°	N	0.12	0.14	0.49	0.77	0°	N	0.00 *	0.01 *	0.04 *	0.75
	45°	S-N	0.89	0.83	0.73	0.56	45°	S-N	0.00 *	0.12	0.38	0.94
	90°	S	0.68	0.70	0.68	n/a	90°	S	0.99	0.98	0.68	0.67
	135°	S-T	0.91	0.85	0.72	0.75	135°	S-T	0.99	0.99	0.99	0.52
	180°	T	0.27	0.63	0.52	0.63	180°	T	0.69	0.60	0.58	0.40
	225°	I-T	0.08	0.13	0.57	0.70	225°	I-T	0.97	0.40	0.62	0.85
	270°	I	0.17	0.48	0.47	0.70	270°	I	0.99	0.77	0.95	0.74
	315°	I-N	0.85	0.85	0.77	0.28	315°	I-N	0.01 *	0.27	0.44	0.95

n/a: not applicable (data was not available). * indicates statistically significant difference.

**Table 5:** The number of eyes that reached a 100% coverage for a diameter of 12 mm, 14 mm, 16 mm, and 18 mm for Caucasian and Asian populations. In each cell, the number of right and left eyes is indicated (OD/OS).

Angular position			**Caucasians**						**Asians**			
			**Distance from the Corneal Centre**						**Distance from the Corneal Centre**			
			**6 mm**	**7 mm**	**8 mm**	**9 mm**			**6 mm**	**7 mm**	**8 mm**	**9 mm**
	0°	N	117/118	102/112	80/94	36/58	0°	N	125/125	116/115	82/96	33/41
	45°	S-N	99/108	60/75	26/29	16/11	45°	S-N	115/125	88/115	27/96	10/41
	90°	S	69/82	40/43	15/15	0/16	90°	S	114/111	79/74	34/33	12/10
	135°	S-T	95/108	65/74	31/33	10/11	135°	S-T	120/120	98/93	57/44	14/18
	180°	T	118/114	95/93	63/56	18/20	180°	T	124/125	120/119	83/93	12/16
	225°	I-T	115/109	82/86	49/50	12/15	225°	I-T	125/125	118/115	97/93	31/33
	270°	I	83/90	54/65	28/34	9/12	270°	I	116/116	86/90	44/52	12/16
	315°	I-N	105/114	79/84	37/49	14/16	315°	I-N	122/123	114/116	73/87	23/33

## References

[1] Efron N., Morgan P.B., Woods C.A., International Contact Lens Prescribing Survey Consortium (2013). An international survey of daily disposable contact lens prescribing. Clin. Exp. Optom..

[2] Haddad M.F., Bakkar M., Gammoh Y., Morgan P. (2016). Trends of contact lens prescribing in Jordan. Contact Lens Anterior Eye.

[3] Holden B.A., Fricke T.R., Wilson D.A., Jong M., Naidoo K.S., Sankaridurg P., Wong T.Y., Naduvilath T.J., Resnikoff S. (2016). Global Prevalence of Myopia and High Myopia and Temporal Trends from 2000 through 2050. Ophthalmology.

[4] Efron N., Morgan P.B., Woods C.A. (2012). International survey of contact lens prescribing for extended wear. Optom. Vis. Sci..

[5] Itoi M., Itoi M., Efron N., Morgan P., Woods C. (2018). Trends in Contact Lens Prescribing in Japan (2003–2016). Contact Lens Anterior Eye.

[6] Vincent S.J. (2018). The rigid lens renaissance: A surge in sclerals. Contact Lens Anterior Eye.

[7] Woods C.A., Efron N., Morgan P. (2020). Are eye-care practitioners fitting scleral contact lenses?. Clin. Exp. Optom..

[8] Consejo A., Llorens-Quintana C., Bartuzel M.M., Iskander D.R., Rozema J.J. (2018). Rotation asymmetry of the human sclera. Acta Ophthalmol..

[9] Ritzmann M., Caroline P.J., Borret R., Korszen E. (2018). An analysis of anterior scleral shape and its role in the design and fitting of scleral contact lenses. Contact Lens Anterior Eye.

[10] DeNaeyer G., Sanders D., van der Worp E., Jedlicka J., Michaud L., Morrison S. (2017). Qualitative Assessment of Scleral Shape Patterns Using a New Wide Field Ocular Surface Elevation Topographer. J. Contact Lens Res. Sci..

[11] Van Nuffel S., Consejo A., Koppen C., Kreps E.O. (2020). The corneoscleral shape in keratoconus patients with and without specialty lens wear. Contact Lens Anterior Eye.

[12] Alonso-Caneiro D., Vincent S.J., Collins M.J. (2016). Morphological changes in the conjunctiva, episclera and sclera following short-term miniscleral contact lens wear in rigid lens neophytes. Contact Lens Anterior Eye.

[13] Consejo A., Bartuzel M.M., Iskander D.R. (2017). Corneo-scleral limbal changes following short-term soft contact lens wear. Contact Lens Anterior Eye.

[14] Consejo A., Behaegel J., Van Hoey M., Wolffsohn J.S., Rozema J.J., Iskander D.R. (2019). Anterior eye surface changes following miniscleral contact lens wear. Contact Lens Anterior Eye.

[15] Walker M.K., Schornack M.M., Vincent S.J. (2020). Anatomical and physiological considerations in scleral lens wear: Conjunctiva and sclera. Contact Lens Anterior Eye.

[16] Hickson-Curran S., Young G., Brennan N., Hunt C. (2016). Chinese and Caucasian ocular topography and soft contact lens fit. Clin. Exp. Optom..

[17] Guillon M., Dumbleton K., Theodoratos P., Patel T., Karkkainen T., Moody K. (2018). Objective Assessment of Ocular Surface Response to Contact Lens Wear in Presbyopic Contact Lens Wearers of Asian Descent. Eye Contact Lens.

[18] Vincent S.J., Alonso-Caneiro D., Collins M.J. (2016). Miniscleral lens wear influences corneal curvature and optics. Ophthalmic Physiol. Opt. J. Br. Coll. Ophthalmic Opt..

[19] Chan S.M., Svitova T.F., Lin M.C. (2017). Accounting for Ethnicity-Related Differences in Ocular Surface Integrity as a Step toward Understanding Contact Lens Discomfort. Eye Contact Lens.

[20] Patel S., Virhia S.K., Farrell P. (1995). Stability of the precorneal tear film in Chinese, African, Indian, and Caucasian eyes. Optom. Vis. Sci..

[21] Cho P., Brown B. (1993). Review of the tear break-up time and a closer look at the tear break-up time of Hong Kong Chinese. Optom. Vis. Sci..

[22] Abass A., Vinciguerra R., Lopes B.T., Bao F., Vinciguerra P., Ambrósio R., Elsheikh A. (2018). Positions of Ocular Geometrical and Visual Axes in Brazilian, Chinese and Italian Populations. Curr. Eye Res..

[23] Blake C.R., Lai W.W., Edward D.P. (2003). Racial and ethnic differences in ocular anatomy. Int. Ophthalmol. Clin..

[24] Hickson-Curran S., Brennan N.A., Igarashi Y., Young G. (2014). Comparative evaluation of Asian and white ocular topography. Optom. Vis. Sci..

[25] Qin B., Tang M., Li Y., Zhang X., Chu R., Huang D. (2012). Anterior Segment Dimensions in Asian and Caucasian Eyes Measured by Optical Coherence Tomography. Ophthalmic Surg. Lasers Imaging Retin..

[26] Consejo A., Llorens-Quintana C., Radhakrishnan H., Iskander D.R. (2017). Mean shape of the human limbus. J. Cataract Refract. Surg..

[27] Abass A., Lopes B.T., Eliasy A., Wu R., Jones S., Clamp J., Ambrósio R., Elsheikh A. (2018). Three-dimensional non-parametric method for limbus detection. PLoS ONE.

[28] Consejo A., Radhakrishnan H., Iskander D.R. (2017). Scleral changes with accommodation. Ophthalmic Physiol. Opt. J. Br. Coll. Ophthalmic Opt..

[29] Abass A., Lopes B.T., Eliasy A., Salomao M., Wu R., White L., Jones S., Clamp J., Ambrósio R., Elsheikh A. (2019). Artefact-free topography based scleral-asymmetry. PLoS ONE.

[30] Iskander D.R., Wachel P., Simpson P.N., Consejo A., Jesus D.A. (2016). Principles of operation, accuracy and precision of an Eye Surface Profiler. Ophthalmic Physiol. Opt. J. Br. Coll. Ophthalmic Opt..

[31] Applegate R.A., Thibos L.N., Twa M.D., Sarver E.J. (2009). Importance of fixation, pupil center, and reference axis in ocular wavefront sensing, videokeratography, and retinal image quality. J. Cataract Refract. Surg..

[32] Kolb H.F.E., Nelson R. (1995). Facts and Figures Concerning the Human Retina.

[33] Gross H. (2005). Handbook of Optical Systems.

[34] Arvo J., David K. (1992). Fast random rotation matrices. Graphics Gems III.

[35] Choi H.J., Lee S.-M., Lee J.Y., Lee S.Y., Kim M.K., Wee W.R. (2014). Measurement of Anterior Scleral Curvature Using Anterior Segment OCT. Optom. Vis. Sci..

[36] Kasahara M., Shoji N., Morita T., Shimizu K. (2014). Comparative optical coherence tomography study of differences in scleral shape between the superonasal and superotemporal quadrants. Jpn. J. Ophthalmol..

[37] Bandlitz S., Baumer J., Conrad U., Wolffsohn J. (2017). Scleral topography analysed by optical coherence tomography. Contact Lens Anterior Eye.

[38] Jeong S., Lemke B.N., Dortzbach R.K., Park Y.G., Kang H.K. (1999). The Asian upper eyelid: An anatomical study with comparison to the Caucasian eyelid. Arch. Ophthalmol..

[39] Most S.P., Mobley S.R., Larrabee W.F. (2005). Anatomy of the eyelids. Facial Plast. Surg. Clin. North Am..

[40] Shaw A.J., Collins M.J., Davis B.A., Carney L.G. (2010). Eyelid pressure and contact with the ocular surface. Investig. Ophthalmol. Vis. Sci..

[41] Song W.C., Kim S.J., Kim S.H., Hu K.S., Kim H.J., Koh K.S. (2007). Asymmetry of the palpebral fissure and upper eyelid crease in Koreans. J. Plast. Reconstr. Aesthetic Surg..

[42] Lam B.L., Lam S., Walls R.C. (1995). Prevalence of Palpebral Fissure Asymmetry in White Persons. Am. J. Ophthalmol..

[43] McCurdy J.A. (2005). Upper blepharoplasty in the Asian patient: The “double eyelid” operation. Facial Plast. Surg. Clin. North Am..

[44] Consejo A., Rozema J.J. (2020). In vivo anterior scleral morphometry, axial length and myopia. Contact Lens Anterior Eye.

[45] Niyazmand H., Read S.A., Atchison D.A., Collins M.J. (2020). Anterior eye shape in emmetropes, low to moderate myopes, and high myopes. Contact Lens Anterior Eye.

[46] Zhao K.K., Yang Y., Wang H., Li L., Wang Z.Y., Jiang F., Qu J.F. (2019). Axial length/corneal radius of curvature ratio and refractive development evaluation in 3- to 4-year-old children: The Shanghai Pudong Eye Study. Int. J. Ophthalmol..

[47] Lam C.S., Goh W.S. (1991). The incidence of refractive errors among school children in Hong Kong and its relationship with the optical components. Clin. Exp. Optom..

[48] Congdon N.G., Youlin Q., Quigley H., Hung P.T., Wang T.H., Ho T.C., Tielsch J.M. (1997). Biometry and primary angle-closure glaucoma among Chinese, white, and black populations. Ophthalmology.

[49] Garaszczuk I.K., Iskander D.R. (2017). Qualitative assessment of tear dynamics with fluorescein profilometry. Contact Lens Anterior Eye.

[50] Zhang X., Li Q., Xiang M., Zou H., Liu B., Zhou H., Han Z., Fu Z., Zhang Z., Wang H. (2013). Bulbar Conjunctival Thickness Measurements with Optical Coherence Tomography in Healthy Chinese Subjects. Investig. Ophthalmol. Vis. Sci..

[51] Walker M.K., Bergmanson J.P., Miller W.L., Marsack J.D., Johnson L.A. (2016). Complications and fitting challenges associated with scleral contact lenses: A review. Contact Lens Anterior Eye.

[52] Kowalski L.P., Collins M.J., Vincent S.J. (2019). Scleral lens centration: The influence of centre thickness, scleral topography, and apical clearance. Contact Lens Anterior Eye.

[53] Consejo A., Behaegel J., Van Hoey M., Iskander D.R., Rozema J.J. (2018). Scleral asymmetry as a potential predictor for scleral lens compression. Ophthalmic Physiol. Opt..

[54] DeNaeyer G., Sanders D.R., Farajian T.S. (2017). Surface coverage with single vs. multiple gaze surface topography to fit scleral lenses. Contact Lens Anterior Eye.

